# Eco-Friendly Ferrimagnetic-Humic Acid Nanocomposites as Superior Magnetic Adsorbents

**DOI:** 10.3390/ma14185125

**Published:** 2021-09-07

**Authors:** Chenhua Deng, Linjie Hou, Caifeng Zhang

**Affiliations:** College of Chemistry, Taiyuan Normal University, Jinzhong 030619, China; houlingjie@sina.cn

**Keywords:** ferrimagnetic nanoparticle, heavy metal, magnetic, adsorption

## Abstract

Recyclable, cheap, eco-friendly, and efficient adsorbent materials are very important for the removal of pollution. In this work, we report the design and implementation of ferrimagnetic-humic acid nanocomposites as superior magnetic adsorbent for heavy metals. Ferrimagnetic and ferrimagnetic-humic acid nanocomposite particles with different morphologies were prepared using the coprecipitation method and hydrothermal synthesis method, respectively. The results show that the morphology of the nanoparticles prepared by the coprecipitation method is more uniform and the size is smaller than that by the hydrothermal synthesis method. Adsorption experiments show that the ferrimagnetic-humic acid nanoparticles prepared by the coprecipitation method has high sorption capacity for cadmium, and the maximum adsorption capacity is about 763 μg/g. At the same time, magnetic technology can be used to realize the recycling of ferrimagnetic-humic acid adsorbents.

## 1. Introduction

Heavy metal pollution in water is related to the health and safety of human beings and other organisms. The heavy metal can destroy photosynthesis, respiration, and enzyme activities of plants. It also affects the development of embryos, organs, and tissues of animals [1,2]. Therefore, how to efficiently remove heavy metals in water is particularly important. In many removal technologies, adsorbent has become the research focus since it has high removal efficiency and does not bring secondary pollution [3,4]. Ferrimagnetic nanoparticles exhibit magnetism and good biocompatibility, which has been widely used in the biomedical, environmental, and material sciences [5,6]. One can use external magnetic fields to separate the nanoparticles rapidly, conveniently, and environmentally, especially as an adsorbent material for the removal of heavy metal pollution. At the same time, nanoparticles have a large specific surface area and high adsorption capacity. However, nanoparticles tend to agglomerate, which will affect their adsorption effect [7,8]. To this end, various methods have been used to introduce kinds of inorganic and organic substances into nanoparticles [9,10].

Humic acid (HA) is a natural organic matter extracted from low-rank coal or plant straw. Humic acid exhibits a macromolecular network structure with a large number of functional groups on the surface, which are dominated by carboxyl and hydroxyl groups [11]. The functional groups on HA can form a relatively stable complex with heavy metal ions, and achieve the goal of removing heavy metals. Moreover, humic acid is environmentally friendly and does not cause secondary pollution during the removal process [12,13]. Therefore, HA is a promising material to modify ferrimagnetic nanoparticles. In addition, ferrimagnetic-HA complexes are also important for the stabilization of HA in the soil, as they inhibit the degradation of HA and extend the turnover time. Recent research indicated that HA has a high affinity to ferrimagnetic nanoparticles, which enhances the stability of nanoparticles dispersion by preventing their aggregation [14]. In addition, the bonding of the nanoparticles to HA can change the surface properties and adsorption properties of the nanoparticles because the adsorption of HA results in polyanionic organic coating on ferrimagnetic nanoparticles. The adsorption capacity for metal cations with complex HA and ferrimagnetic nanoparticles was reported to be larger than that with the respective HA or ferrimagnetic nanoparticles alone [15]. Shitong Yang et al. [16] prepared the core-shell structure of ferrimagnetic-humic acid nanoparticles by chemical co-precipitation method, which can effectively remove the radioactive pollutant Eu (III) with a removal efficiency of 99%. C. J. Zhou et al. [17] synthesized humic acid-coated iron oxide nanoparticles used to rapidly and efficiently remove methylene blue and rhodamine organic dyes. Therefore, the ferrimagnetic nanoparticles which are combined with HA may be able to synergistically remove heavy metal pollution.

To prepare ferrimagnetic-humic acid nanocomposite particles, the chemical coprecipitation method is widely used at present. For nanomaterials, we know that their properties are largely dependent on the morphology and size of the nanostructures. In this paper, ferrimagnetic-humic acid nanocomposite particles with different morphologies were prepared using different preparation methods. To obtain the optimal adsorption conditions of ferrimagnetic-humic acid nanocomposite particles on heavy metals, the relationship between the morphology of nanoparticles and the adsorption effect was discussed.

## 2. Materials and Methods

### 2.1. Preparation

The ferrimagnetic nanoparticles were fabricated using coprecipitation method and hydrothermal synthesis method, respectively. For the coprecipitation method, 6.0 g FeCl_3_·6H_2_O and 4.2 g FeSO_4_·7H_2_O was dissolved in 100 mL deionized water, and heated to 90 °C. Then, 10 mL 25% ammonium hydroxide was added into the solution and humic acid sodium salt, dissolved in water, were added rapidly and sequentially. The above mixture was continually stirred at 90 °C for 30 min and then cooled to room temperature. Finally, the mixture was filtrated, and washed with distilled water and ethanol three times, respectively. The black powder was collected and dried in an oven (BPG-9140A, Shanghai Yiheng Scientific Instrument Co., LTD, Shanghai, China) at 50 °C. For the hydrothermal synthesis method, 0.67 g FeCl_3_·6H_2_O was dissolved in 20 mL ethylene glycol completely, and 1.8 g sodium acetate and 0.07 g sodium polyacrylate were added into the solution. Then, the mixture solution was stirred for 30 min, and transferred into a 25 mL Teflon-lined stainless-steel autoclave (Binhai County zhengxin instrument Factory, Yancheng, Jiangsu Province, China). The reaction was processed under 200 °C for 12 h. Next, the obtained black powder was washed with distilled water and ethanol three times, respectively, as before. The ferrimagnetic-HA composite nanoparticle was prepared using the same method as ferrimagnetic nanoparticles, and just HA was added. All the reagents used were purchased from Aladdin Ltd., shanghai, China, and were used as received without further purification.

The HA used were from the Humic Acid Quality Testing Center of the China Humic Acid Industry Association, Jinzhong, China.

### 2.2. Characterization

The Fourier transform infrared spectroscopy (FTIR) of nanoparticles was detected by an infrared spectrometer (Nicolet Is5, Thermo Electron Corporation, Waltham, MA, USA) in the range of 400–4000 cm ^−^^1^. Thermal gravimetric analysis (TG) and differential thermal analysis (DTA) were recorded using a thermal heavy analyzer (STA 2500 Regulus, Netzsch, Bavaria, Germany) in the range of 0–800 °C. X-ray diffraction (XRD) patterns were estimated using a PANalytical X′ Pert Pro X-ray diffractometer (PANalytical B.V., Almelo, the Netherlands) in the θ–2θ configuration with the Cu *k*_a_ X-ray radiation. The morphology and lattice structure of ferrimagnetic and ferrimagnetic-humic acid nanoparticles fabricated using different method were carried out by a JEOL JSM-7500F cold field scanning electron microscopy (SEM, JEOL, Tokyo, Japan) and a FEI Tecnai G^2^ F20 S-TWIN transmission electron microscope (TEM, FEI, Hillsborough, OR, USA). The magnetic properties were measured using the quantum design physical property measurement system with a vibrating sample magnetometer (VSM) accessory (squid-VSM, Quantum Design INC, San Diego, CA, USA). The removal efficiency was characterized by inductively coupled plasma atomic emission spectrometry (ICP, ICAP RQ, Thermo Fisher Scientific, Waltham, MA, USA). We choose Cd as the representative element of heavy metal pollution in water to carry out the adsorption experiment.

### 2.3. Heavy Metal Adsorption Experiment

A total of 20 mL of Cd^2+^ standard solutions with different concentrations (0.05 mg/L, 0.10 mg/L, 0.15 mg/L, 0.20 mg/L and 0.25 mg/L) was accurately measured, and 10 mg of the adsorbent samples prepared in the above steps were added. The pH value of the solution was adjusted accurately with the help of a pH meter (FE28, Mettler Toledo Instruments (Shanghai) Co., LTD, Shanghai, China). Then, at the same oscillation speed, the solution was placed in a thermostatic oscillator (SHY-2A, Changzhou Guoyu Instrument Manufacturing Co., LTD, Changzhou, Jiangsu, China) for adsorption experiment at room temperature lasting 30 min. After the reaction, the adsorbent in the mixture was separated by a permanent magnet. The supernatant was taken and the concentration of the remaining Cd^2+^ ions in the solution was determined by ICP.

## 3. Results

### 3.1. Characterization of Ferrimagnetic and Ferrimagnetic-HA

**Figure 1:**
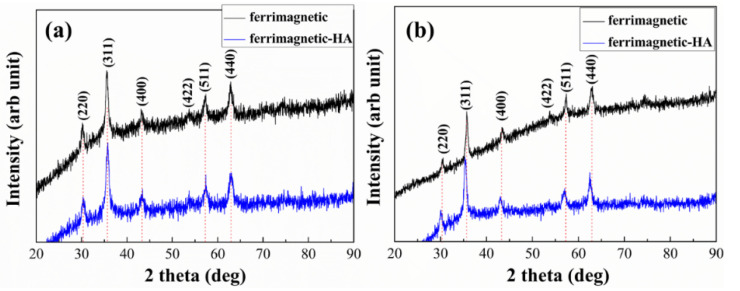
X-ray diffraction patterns of the ferrimagnetic and ferrimagnetic-HA nanoparticles prepared using (**a**) the coprecipitation method and (**b**) the hydrothermal synthesis method.

Figure 1 presents the powder X-ray diffraction patterns of the ferrimagnetic and ferrimagnetic-HA nanoparticles prepared using (a) the coprecipitation method and (b) the hydrothermal synthesis method. The characteristic XRD peaks of all as-synthesized samples have nearly identical peak positions. Ferrimagnetic phase with inverse spinel structure (JCPDS 19-0629) was obtained by the coprecipitation method and hydrothermal synthesis method. The obtained ferrimagnetic-HA nanoparticles show only the similar peaks with ferrimagnetic nanoparticles, without any other peaks, indicating that the structure of the ferrimagnetic nanoparticles was not changed after modification with HA. Figure 1 shows that no other peaks related to impurities were detected, which confirms that the synthesized products are of high purity and with good crystallinity. We calculated the size of the particles by the Sherrer equation. The average diameter of the ferrimagnetic nanoparticle prepared by the coprecipitation and hydrothermal synthesis method is about 16 nm and 19 nm, respectively. For both samples, the HA modified ferrimagnetic nanoparticles showed a smaller particle size with about 15 nm and 14 nm, respectively, which indicated that HA efficiently reduces their aggregation.

**Figure 2:**
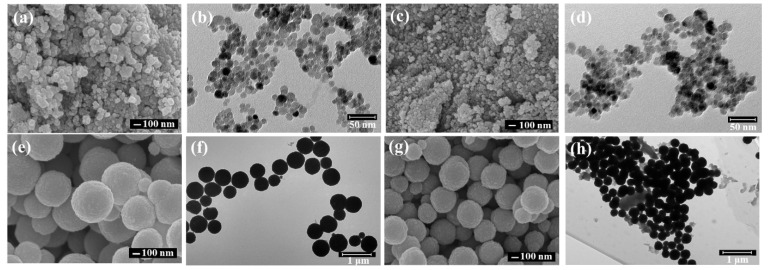
SEM and TEM images of (**a**,**b**) the ferrimagnetic and (**c**,**d**) ferrimagnetic-HA nanoparticles prepared using the coprecipitation method, and (**e**,**f**) the ferrimagnetic and (**g**,**h**) ferrimagnetic-HA nanoparticles prepared using the hydrothermal synthesis method.

The morphologies of the as-synthesized nanoparticles were detected by SEM and TEM. Figure 2 shows the surface morphologies of the prepared (Figure 2a,b) ferrimagnetic and (Figure 2c,d) ferrimagnetic-HA nanoparticles using the coprecipitation method. It can be seen that the nanoparticles showed a homogenously spherical-shape. The average diameter of the ferrimagnetic nanoparticles is about 35 nm with a smooth surface. The size of the nanoparticles was reduced to about 10 nm under TEM observation, which is in agreement with the result of XRD analysis. Moreover, the morphologies of the nanoparticles do not show significant change after coating HA. This suggests that the addition of HA does not alter the microscopic morphology of the nanoparticles. Figure 2 shows the surface morphologies of the prepared ferrimagnetic (Figure 2e,f) and ferrimagnetic-HA nanoparticles (Figure 2g,h) using the hydrothermal synthesis method. Compared with the nanoparticles obtained by the coprecipitation method, the homogeneity of the nanoparticles was improved, and the morphology of the nanoparticles does not alter after HA addition. The size of the nanoparticles become larger with a diameter of about 400 nm fabricated by the hydrothermal method, which is quite different from the XRD results. This may be due to that the size of the nanoparticles prepared by the hydrothermal synthesis method is relatively large, which is not accurate to calculate the particle size by using the Sherrer formula. The ferrimagnetic-HA nanoparticles with different sizes, were obtained using the two mostly common preparation methods. The HA modified ferrimagnetic nanoparticles have smaller particle size and show good dispersion, which indicated HA efficiently reduces their aggregation.

**Figure 3:**
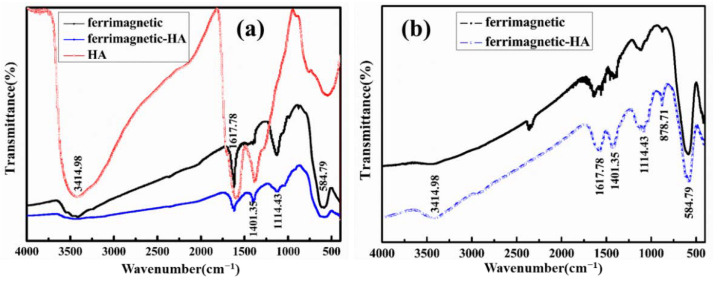
FTIR spectra of HA, ferrimagnetic and ferrimagnetic-HA nanoparticles prepared using (**a**) the coprecipitation method and (**b**) the hydrothermal synthesis method.

FTIR is an established tool to identify functional groups of molecules and can analyze the surface groups of the samples. Figure 3a exhibits FTIR spectra of HA, ferrimagnetic and ferrimagnetic-HA nanoparticles prepared using the coprecipitation method. The absorption peak at 584 cm^−1^ was attributed to the characteristic absorption of the stretching vibration of Fe-O bond. The absorption peak of 1114 cm^−1^ came from the hydroxyl group. All samples showed the absorption peaks of 1617 cm^−1^ and 3414 cm^−1^, which may be due to the vibration of adsorbed water molecules. Compared with ferrimagnetic nanoparticles, the composite nanoparticle appeared the absorption peak of C=O stretches at 1401 cm^−1^, indicating that the humic acid has been successfully bonded onto the ferrimagnetic nanoparticles [18]. Figure 3b shows FTIR spectra of ferrimagnetic and ferrimagnetic-HA nanoparticles prepared using the hydrothermal synthesis method. It can be seen that the nanoparticles exhibit the same absorption peaks as the one prepared using the coprecipitation method. Furthermore, the ferrimagnetic-HA nanoparticles showed an absorption peak of C=O that stretches at 1401 cm^−1^. The above results indicate that HA has been successfully bonded onto the ferrimagnetic nanoparticles prepared by the two methods.

**Figure 4:**
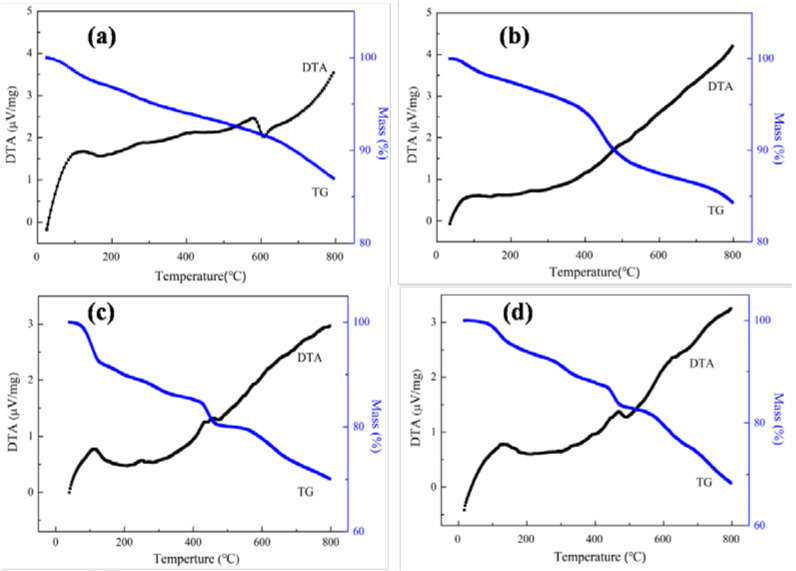
TG and DTA curves of (**a**) ferrimagnetic and (**b**) ferrimagnetic-HA nanoparticles prepared using the coprecipitation method, and (**c**) ferrimagnetic and (**d**) ferrimagnetic-HA nanoparticles prepared using the hydrothermal synthesis method.

To obtain the thermal stability of the nanoparticles, thermogravimetric analysis was performed under a nitrogen atmosphere, and the heating rate was 10 °C/min. Figure 4 presents the TG and DTA curves of ferrimagnetic and ferrimagnetic-HA nanoparticles prepared using the coprecipitation method and hydrothermal synthesis method. It can be seen that the mass of all samples decreases with the increase of temperature, which mainly comes from the adsorbed water and solvents during the preparation process. The weight loss at temperatures ranging from 30 °C to 100 °C is mainly due to moisture and adsorbed gases. The mass loss near 400 °C is mainly caused by the phase transformation of ferrimagnetic nanoparticles, that is, Fe_3_O_4_ is transformed into α-Fe_2_O_3_. Compared with ferrimagnetic nanoparticles, the hybrid nanocomposites exhibit greater weightlessness between 400 and 500 °C. This may be attributed to the contribution of decarboxylation and pyrolysis of aromatic nucleus of the condensed ring in HA.

**Figure 5:**
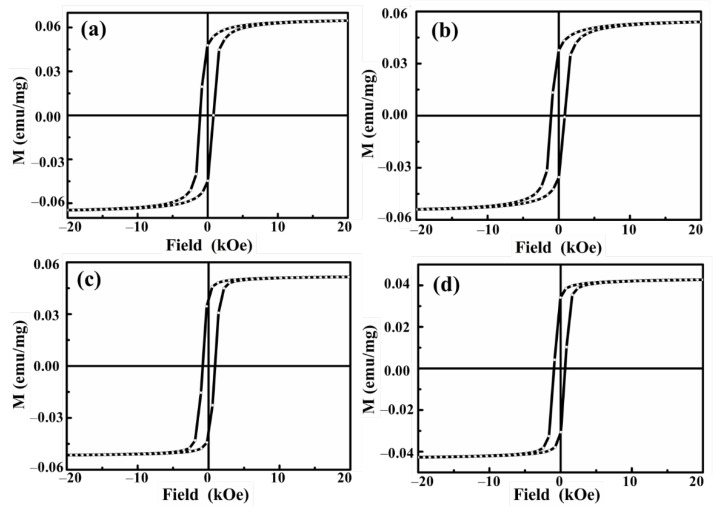
Magnetic hysteresis loops of (**a**) ferrimagnetic and (**b**) ferrimagnetic-HA nanoparticles prepared using the coprecipitation method, and (**c**) ferrimagnetic and (**d**) ferrimagnetic-HA nanoparticles prepared using the hydrothermal synthesis method measured at room temperature.

The magnetic hysteresis loops were measured at room temperature for ferrimagnetic and ferrimagnetic-HA nanoparticles prepared using the coprecipitation method and hydrothermal synthesis method, as shown in Figure 5. It can be seen that all samples exhibit hysteresis at room temperature. The coercivity (H_c_) for ferrimagnetic nanoparticle prepared using the coprecipitation method is about 1018 Oe, and the H_c_ for ferrimagnetic nanoparticle prepared using the hydrothermal synthesis method is about 834 Oe. The size of the nanoparticles obtained by the coprecipitation method is smaller than that obtained by the hydrothermal synthesis method. Moreover, the magnetic moment of the ferrimagnetic nanoparticles prepared using the hydrothermal synthesis method is also smaller than that of the coprecipitation method. The nanoparticles with small sizes exhibit strong magnetization and coercivity. With the size of grain particles reducing, the magnetic properties of the materials are strongly affected by the size. This is due to the influence of the thermal energy over the magnetic moment ordering, which originates from the paramagnetic relaxation phenomenon. For both samples, the magnetization gets smaller due to the addition of nonmagnetic substances, and the coercivity is essentially unchanged when the HA is coated onto the nanoparticles. Although magnetization strength decreased after HA addition, it could be inferred that the separation of ferrimagnetic-HA nanoparticles in aqua media could be controlled by magnetic fields with its magnetization strength. From the observed behavior, the prepared nanoparticles have large magnetization and coercivity, which can exhibit a high stability and strong response to the external magnetic field. This is very important in the application of magnetic separation technology to eliminate heavy metal pollutants.

### 3.2. Adsorption Effect of Nanocomposites on Pollutant

**Figure 6:**
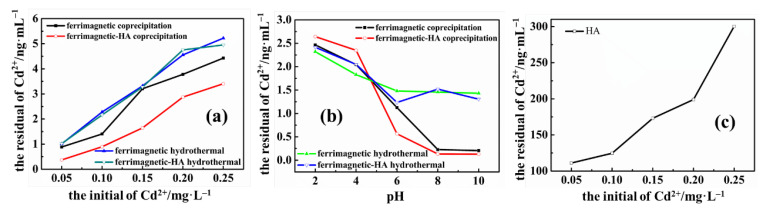
The effects of (**a**) initial heavy metal concentration (pH = 6; adsorbent quality 10 mg; contact time 30 min; 25 °C), (**b**) solution pH (Cd^2+^ initial concentration 0.1 mg/L; adsorbent quality 10 mg; contact time 40 min; 25 °C) on the adsorption efficiency for ferrimagnetic and ferrimagnetic-HA nanocomposites, and (**c**) the effect of initial heavy metal concentration on the adsorption efficiency for HA only.

To obtain the adsorption effect of nanoparticles on heavy metals, the concentration of Cd^2+^ in water was analyzed by ICP. The main parameters that affect the binding of heavy metal ions to ferrimagnetic-HA nanoparticles are the concentration of metal ions, pH and other parameters. Therefore, the effects of the initial heavy metal concentration and solution pH on the removal of heavy metal ions by ferrimagnetic-HA nanoparticles and HA were investigated, respectively. As shown in Figure 6a, the concentration of the remaining Cd^2+^ in the solution is increased with the increase of the initial concentration of Cd^2+^. The reason is that when the number of ferrimagnetic-HA nanoparticles remains the same, the number of active sites on the surface of ferrimagnetic-HA nanoparticles is also constant. With the increase of the amount of Cd^2+^, the active sites on the surface are gradually occupied by Cd^2+^, which leads to the decrease of the adsorption effect. In addition, the pH of the solution is also an important factor affecting the adsorption effect, which can be seen in Figure 6b. The initial concentration of Cd^2+^ was 0.10 mg/L. With the increase of pH, the concentration of residual Cd^2+^ in the solution gradually decreases and tends to remain constant. Moreover, the adsorption effect of the adsorbents prepared by the coprecipitation method is better than that prepared by the hydrothermal synthesis method. This result is consistent with the previous morphological analysis results. As can be seen from Figure 2, the size of nanoparticles prepared by the coprecipitation method is smaller than that prepared by the hydrothermal synthesis method. At this time, the number of active sites on the surface increased, and thus, the nanocomposite prepared by the coprecipitation method has a high adsorption effect. Figure 6c exhibits the effect of the initial heavy metal concentration on the adsorption efficiency for HA only. It can be seen that HA also has a certain adsorption capacity for heavy metal. When ferrimagnetic nanoparticles and HA were combined, the adsorption effect of nanocomposites was stronger than that of HA and ferrimagnetic nanoparticles separately. As a result, the combination of HA and ferrimagnetic nanoparticles can significantly enhance the adsorption effect of the adsorbent, and achieve the purpose of synergistic removal of heavy metals. This can be attributed to the fact that HA has a large number of active functional groups on the surface, so it can be further complexed with heavy metals to improve the adsorption effect.

**Figure 7:**
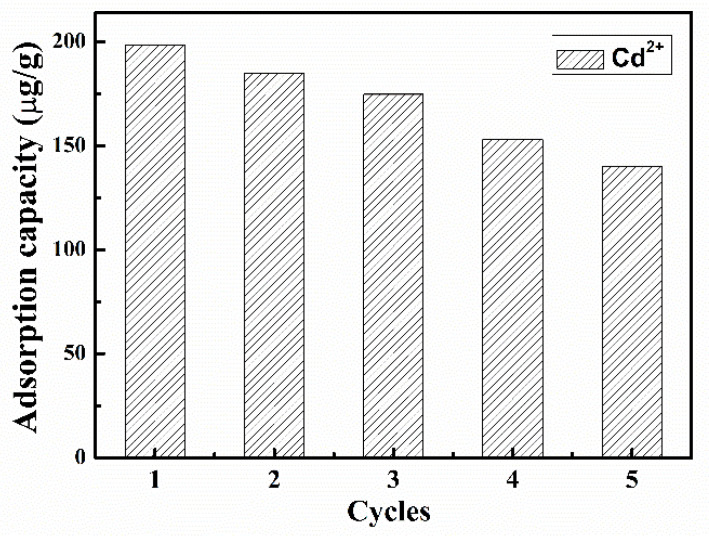
Reusability of ferrimagnetic-HA nanoparticles in the removal of Cd^2+^ for five consecutive adsorption–desorption cycles. The initial concentrations of Cd^2+^ was 0.1 mg/L. The adsorbent dosage was 10 mg. The contact time was 30 min.

Regeneration and reuse of adsorbent are essential for economic use. For ferrimagnetic-HA nanocomposite particles, magnetic technology can be used to realize the recycling of adsorbents. In this study, desorption behavior was studied using 0.03 M of EDTA solution, as shown in Figure 7. The adsorption capacity of Cd^2+^ decreased gradually with the increase of regeneration cycle. After the fifth cycle, the loaded amount of Cd^2+^ was 70.6% of the amount by the fresh adsorbent, demonstrating that ferrimagnetic-HA nanoparticles could be regenerated and reused using EDTA.

## 4. Discussion

According to the above discussion, the nanoparticles have similar crystal structure and properties prepared by the coprecipitation and hydrothermal synthesis method, respectively. However, the size of the nanoparticles is different. In this regard, the size effect of nanoparticle on the adsorption performance can be compared well. In comparison, the size of the nanoparticles prepared by the coprecipitation method is smaller. At the same time, the adsorption experiments show that the nanocomposite particles prepared by the coprecipitation method have greater adsorption capacity (Figure 6).

To further gain insight into the adsorption mechanism of the nanocomposite particles, the adsorption isotherms of Cd^2+^ on magnetic ferrimagnetic-HA nanocomposites obtained by the coprecipitation method were fitted according to Langmuir and Freundlich equations, respectively [9]. First, the adsorption of Cd^2+^ ions onto the ferrimagnetic-HA nanocomposites with pH = 6 was investigated as a function of the initial heavy metal concentration for 30 min at 25 °C, which can be seen from Figure 6a. Then, the adsorption results were analyzed as follows:

$$\mathrm{Equilibrium}\;\mathrm{absorption}\;\mathrm{capacity}:\;q_{e}=\frac{\left(c_{0}-c_{e}\right)\cdot V}{w}$$

where *q_e_* is the equilibrium adsorption capacity (µg/g) of ferrimagnetic-HA nanocomposite at a contact time of 30 min; *c_0_* and *c_e_* are the initial mass concentration and equilibrium mass concentration (ng/mL) of Cd^2+^, respectively; *V* is the volume of solution (20 mL); *w* is the mass of the magnetic composite (10 mg).

$$\mathrm{Langmuir}\;\mathrm{equation}:\;\frac{1}{q_{e}}=\frac{1}{k_{L}q_{m}}\times \frac{1}{c_{e}}+\frac{1}{q_{m}}$$

where *q_e_* is the equilibrium adsorption capacity (µg/g); *c_e_* is the equilibrium mass concentration (ng/mL) of Cd^2+^; *K_L_* is Langmuir adsorption constant (mL/ng); *q_m_* is the maximum adsorption capacity (µg/g).

**Figure 8:**
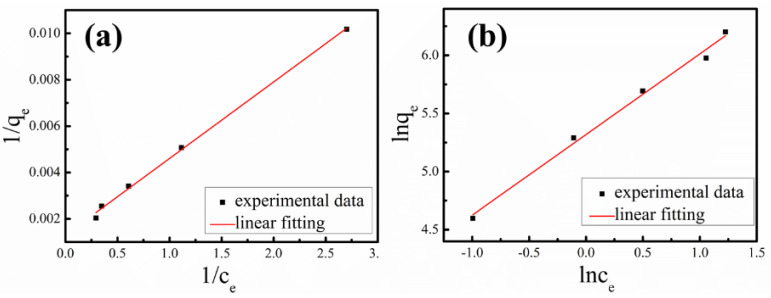
Cd^2+^ adsorption isotherm on the ferrimagnetic-HA nanocomposites prepared by the coprecipitation method, with (**a**) Langmuir Equation fitting and (**b**) Freundlich equation fitting.

1/*q_e_* was plotted against 1/*c_e_* (Figure 8a), and the maximum sorption capacity *q_m_* can be obtained from the linear fitting result:

$$\mathrm{Freundlich}\;\mathrm{equation}:\;lnq_{e}=lnK_{F}+\frac{1}{n}lnc_{e}$$

where *K_F_* is the adsorption constant of Freundlich equation; *n* is the Freundlich equation index. *lnq_e_* was plotted against *lnc_e_*, which can be seen in Figure 8b.

According to the fitting results, the Langmuir isotherm model with a correlation coefficient as high as 0.9974 better represents the equilibrium adsorption of Cd^2+^ on ferrimagnetic-HA nanocomposite as compared with the Freundlich equation. In this regard, the adsorption process belongs to the monolayer coverage of Cd^2+^ on the surface of ferrimagnetic-HA nanocomposite with uniform adsorption site energy. The maximum sorption capacity *q_m_* of ferrimagnetic-HA nanocomposite prepared by the coprecipitation method is about 763 μg/g, which is not large compared with other reported magnetic adsorbents [19]. However, the initial concentration of Cd^2+^ ion reported in other papers is relatively large, such as ferrimagnetic chitosan-phenylthiourea resin, for which the initial concentration of Cd^2+^ ion is 100 mg/L [20], magnetic graphene oxide nanocomposite, for which the initial concentration of Cd^2+^ ion is 200 mg/L [21], and Fe_3_O_4_/SiO_2_-GO, for which the initial concentration of Cd^2+^ ion is 100 mg/L [22]. The initial concentration of Cd^2+^ we used was 0.05–0.25 mg/L, and generally 0.1 mg/L. This result indicates that the ferrimagnetic-HA nanocomposite prepared by the coprecipitation method possess strong adsorption capability towards Cd^2+^ with a small initial concentration, which is closer to the actual situation.

The M_s_ of the ferrimagnetic-HA nanocomposite prepared by the coprecipitation method is about 52 emu/g, which is comparable to the data reported in the literature [23,24]. For ferrimagnetic-HA nanocomposite particles, magnetic technology can be used to realize the recycling of adsorbents. At the same time, both ferrimagnetic nanoparticles and humic acid are environmentally friendly. Thus, ferrimagnetic-humic acid nanocomposites can be utilized as a superior adsorbent for practical adsorption. In addition, the nanocomposites may also exhibit adsorption properties for other heavy metals and organic dyes.

## 5. Conclusions

Ferrimagnetic and ferrimagnetic-HA nanoadsorbents with different sizes were prepared by the coprecipitation method and hydrothermal synthesis method, respectively. In comparison, the nanoparticles prepared by the coprecipitation method have uniform morphology, and the size is only about 35 nm with smooth surface. The FTIR results show that HA has been successfully compounded onto ferrimagnetic nanoparticles by using both methods. Moreover, for the composite nanoparticles, HA does not change the morphology and structure of ferrimagnetic nanoparticles. Adsorption experiments show that the adsorption effect of ferrimagnetic-HA nanocomposite particles prepared by the coprecipitation method is good, and the maximum adsorption capacity is as high as 763 μg/g. At the same time, magnetic technology can be used to realize the recovery of composite adsorbents. The adsorbent can also be used to remove other heavy metals and organic pollutants.

## Data Availability

Not applicable.
